# Supplementary material for: Clinical and Immunological Impact of Ocrelizumab Extended Interval Dosing in Multiple Sclerosis: A Single-Center, Real-World Experience
Source: Int J Mol Sci. 2024 May 14;25(10):5353. doi: 10.3390/ijms25105353 (PMC11121257; doi:10.3390/ijms25105353)

Correlation Matrix

		Age	N previous DMT	Disease Duration	Baseline ARR	Baseline EDSS	Baseline CD19+	Baseline CD8+	Baseline CD4+	Baseline Lymphocyte count	Baseline igG	Baseline IgA	Bas I
Age	Spearman's rho	—											
	df	—											
	p-value	—											
N previous DMT	Spearman's rho	-0.073	—										
	df	91	—										
	p-value	0.48814	—										
Disease Duration	Spearman's rho	0.249 *	0.680 ***	—									
	df	91	91	—									
	p-value	0.01598	<.00001	—									
Baseline ARR	Spearman's rho	-0.380 ***	-0.225 *	-0.544 ***	—								
	df	91	91	91	—								
	p-value	0.00017	0.03004	<.00001	—								
Baseline EDSS	Spearman's rho	0.458 ***	0.247 *	0.314 **	-0.372 ***	—	0.078	0.059	0.102	-0.017			
	df	91	91	91	91	—	91	91	91	91			
	p-value	<.00001	0.01721	0.00220	0.00024	—	0.45552	0.57320	0.33082	0.87327			
Baseline CD19+	Spearman's rho	0.007	-0.001	-0.006	-0.173		—						
	df	91	91	91	91		—						
	p-value	0.94379	0.99004	0.95483	0.09706		—						
Baseline CD8+	Spearman's rho	-0.184	-0.098	-0.078	0.071		0.300 **	—					
	df	91	91	91	91		91	—					
	p-value	0.07706	0.34935	0.45501	0.50076		0.00352	—					
Baseline CD4+	Spearman's rho	0.066	-0.191	-0.106	-0.036		0.447 ***	0.431 ***	—				
	df	91	91	91	91		91	91	—				
	p-value	0.52740	0.06598	0.31013	0.73242		<.00001	0.00002	—				
Baseline Lymphocyte count	Spearman's rho	-0.103	-0.205 *	-0.204	0.100		0.426 ***	0.536 ***	0.664 ***	—			
	df	91	91	91	91		91	91	91	—			
	p-value	0.32485	0.04873	0.05005	0.34260		0.00002	<.00001	<.00001	—			
Baseline igG	Spearman's rho	-0.101	-0.004	-0.122	-0.050	0.025	0.077	-0.064	-0.019	0.093	—		
	df	91	91	91	91	91	91	91	91	91	—		
	p-value	0.33495	0.96669	0.24440	0.63250	0.81063	0.46304	0.54048	0.85447	0.37561	—		
Baseline IgA	Spearman's rho	0.079	0.136	0.099	-0.245 *	0.368 ***	-0.043	-0.008	-0.033	-0.057	0.439 ***	—	
	df	91	91	91	91	91	91	91	91	91	91	—	
	p-value	0.45448	0.19349	0.34291	0.01799	0.00029	0.68246	0.94084	0.75183	0.58837	0.00001	—	
Baseline IgM	Spearman's rho	0.010	-0.124	-0.208 *	-0.064	-0.026	-0.151	-0.059	-0.018	-0.035	0.413 ***	0.328 **	
	df	91	91	91	91	91	91	91	91	91	91	91	
	p-value	0.92272	0.23668	0.04593	0.54386	0.80827	0.14783	0.57219	0.86274	0.74263	0.00004	0.00132	

Note. * p < .05, ** p < .01, *** p < .001

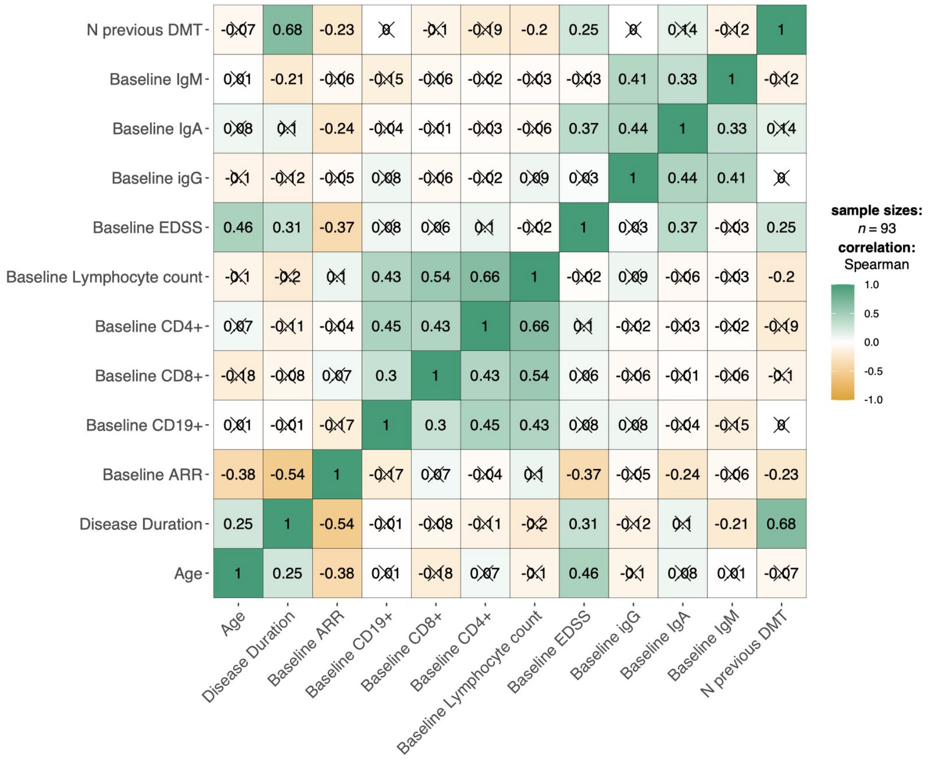

Supplement: Supplementary file 1 [file ijms-25-05353-s001.zip › Figure S1. Correlation matrix and chart for baseline characteristics.pdf]
