# Supplementary material for: Clinical and Immunological Impact of Ocrelizumab Extended Interval Dosing in Multiple Sclerosis: A Single-Center, Real-World Experience
Source: Int J Mol Sci. 2024 May 14;25(10):5353. doi: 10.3390/ijms25105353 (PMC11121257; doi:10.3390/ijms25105353)
Supplement: Supplementary file 1 [file ijms-25-05353-s001.zip › OCR_Supplementary tables.docvx.pdf]

SUPPLEMENTARY TABLES

	**CD19+<10 cells/µL (depleted)**	**CD19+≥10 cells/µL (non-depleted)**	**P value**
**Clinical activity**	2 (0.8%)	0 (0 %)	0.99
**MRI activity**	8 (3.9%)	4 (4.3%)	0.90
**CDP**	17(6.6%)	3 (7.5%)	0.74
**Loss of NEDA3**	36 (14.1%)	3 (7.5%)	0.32

Supplementary Table S1. Clinical and radiological outcomes in B-depleted vs non-depleted subjects. Number of events and percentage per-group. *(Fisher's exact test)*

	**SID**	**EID**	**P-value**
**Low igG**	19 (9.4%)	11 (12.0%)	0.53
**Low igM**	28 (13.8%)	21 (22.8%)	0.06
**Low igA**	3 (1.5)	2 (2.2)	0.64

Supplementary Table S2. Incidence of hypogammaglobulinemia in SID and EID. Number of events and percentage per-group. *(Fisher's exact test)*
