# Supplementary figures and images for: Clinical and Immunological Impact of Ocrelizumab Extended Interval Dosing in Multiple Sclerosis: A Single-Center, Real-World Experience
Source: Int J Mol Sci. 2024 May 14;25(10):5353. doi: 10.3390/ijms25105353 (PMC11121257; doi:10.3390/ijms25105353)

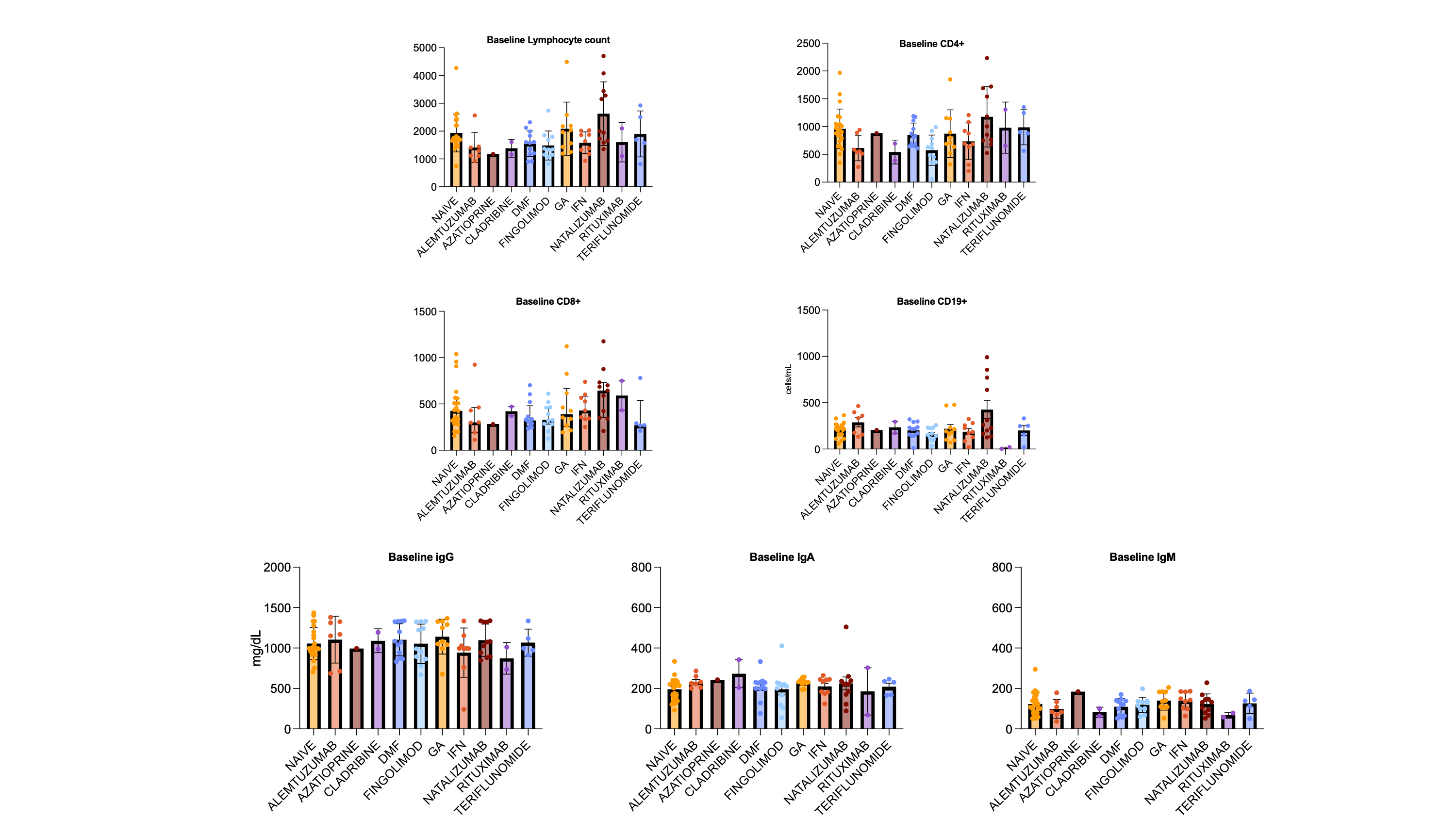

Supplement: Supplementary file 1 [file ijms-25-05353-s001.zip › Figure S2. Lymphocytes count and immunoglobulin levels at baseline grouped by previous DMT..tiff]
